# The Application of Ultrasonography in the Detection of Airway Obstruction: A Promising Area of Research or Unnecessary Gadgetry?

**DOI:** 10.3390/life15071003

**Published:** 2025-06-24

**Authors:** Sabina Kostorz-Nosal, Mariusz Kowaliński, Aleksandra Spyra, Bartłomiej Gałuszka, Szymon Skoczyński

**Affiliations:** Department of Lung Diseases and Tuberculosis, Faculty of Medical Sciences in Zabrze, Medical University of Silesia in Katowice, 41-803 Zabrze, Poland; kowalinski.mariusz@gmail.com (M.K.); aleksandra1828@gmail.com (A.S.); bartlomiejgaluszka.lote@gmail.com (B.G.); sz.skoczynski@sum.edu.pl (S.S.)

**Keywords:** airway obstruction, chronic obstructive pulmonary disease, diaphragmatic mobility, lung cancer, ultrasonography

## Abstract

Since the COVID-19 pandemic, the utilization of transthoracic ultrasonography (TTU) in the evaluation of pulmonary field artefacts has become standard practice among clinicians. However, there is a considerable lack of knowledge regarding the assessment of diaphragm mobility in the context of various lung diseases. Although numerous conditions are known to affect diaphragm mobility, including neurological, cardiovascular, and infectious diseases, it appears that pulmonary diseases may also limit the mobility of this major respiratory muscle. Despite the evidence of diaphragm mobility disorders in patients diagnosed with lung cancer, there is a discrepancy in the literature regarding the function of the diaphragm in individuals with chronic obstructive pulmonary disease (COPD). A shared aetiological factor frequently results in the co-occurrence of the aforementioned diseases. It is, however, possible to detect patients whose obstructive airway disease is caused only by the compression of infiltrative and nodal lesions rather than COPD. Bilateral TTU of diaphragmatic mobility in correlation with other available pulmonary function tests and radiological imaging may prove to be a valuable approach to isolating lung cancer patients with COPD overdiagnosis. Conversely, the overdiagnosis of COPD has been implicated in the potentially unnecessary and harmful use of inhaled medications with their adverse effects (e.g., cardiac arrhythmias, limb tremor, cough, and pneumonia), the failure to decrease obstruction in cases of other lung disorders, and the potential to contribute to the delayed diagnosis of the underlying condition responsible for the respiratory symptoms. This paper aims to provide a comprehensive overview of the utilization of ultrasound in the evaluation of diaphragm movement impairments for the detection of obstructions while also delineating the underlying limitations of this technique. Moreover, we propose a diagnostic algorithm for the purpose of excluding unilateral obstruction resulting from infiltrative neoplastic masses based on the ultrasound assessment of diaphragmatic mobility.

## 1. Introduction

Airway obstruction is defined as the complete or partial narrowing of the airways regardless of aetiology, resulting in impaired ventilation. When considering chronic obstructive pulmonary disease (COPD), airway narrowing affects small airways, resulting in an increase in total resistance [1,2]. It is indisputable that the most prevalent cause of chronic airflow obstruction on a global scale is smoking and exposure to air pollution [1,3,4]. These circumstances, in conjunction with the presence of allergenic agents, may result in inflammation of the airways, leading to swelling of the airway mucosa, remodelling of the bronchial wall, and fibrosis. Secondly, airway obstruction may be caused by the presence of excessive mucus within the respiratory tract. This condition may be observed in individuals with either abnormal mucus composition (e.g., cystic fibrosis) or impaired ciliary transport (e.g., ciliary dyskinesia) [5,6]. Finally, fine bronchioles are physiologically suspended in the interstitial tissue of the lung, thus supporting the peripheral airway and providing the lung with its elasticity. Consequently, the narrowing of the airway may also be attributed to the presence of developmental abnormalities, bronchomalacia, external compression, or pathological conditions affecting the bronchial lumen [7,8].

Irrespective of the underlying cause, airway narrowing results in impaired ventilation. Depending on the severity of the disease, patients may experience debilitating symptoms, including cough, dyspnoea, and fatigue, which can lead to a reduction in physical activity. This, in turn, can have a direct impact on the quality of life of the patient and may also impair their performance status [9,10,11]. In light of the aforementioned evidence, the diagnosis of airway obstruction remains a fundamental aspect of the therapeutic process.

In accordance with the latest GOLD guidelines (the Global Initiative for Chronic Obstructive Lung Disease), the presence of the FEV1/FVC ratio (forced expiratory volume in 1 s/forced vital capacity) to a level below 70% after the administration of a bronchodilator is regarded indicative of obstruction and authorizes a diagnosis of COPD [12]. However, a recent meta-analysis revealed that depending on the diagnostic criteria that are adopted, the prevalence of COPD is overdiagnosed by 42% according to the GOLD definition and by 48.2% when the LLN (the lower limit of normal) is adopted [13]. In light of the aforementioned guidelines, the diagnosis of COPD frequently necessitates the implementation of inhalation therapy [12]. Among patients with a false positive diagnosis of COPD, 47.5% use inhaled medication [14]. As a consequence, the overdiagnosis of COPD has been implicated in the potentially unnecessary and harmful use of inhaled medications with their adverse effects (e.g., cardiac arrhythmias, limb tremor, cough, and pneumonia), the failure to decrease obstruction in cases of other lung disorders, and the potential to contribute to delayed diagnosis of the underlying condition responsible for the respiratory symptoms [13].

The rationale behind these actions is elucidated on the molecular level. The location of B2-adrenergic receptors (β2AR) was determined to be within the upper and lower respiratory tracts, with a notable concentration observed in the alveolar walls [15]. The function of β2AR has been demonstrated to be implicated in numerous physiological processes, encompassing not only bronchodilation but also vasodilatation, mucociliary clearance, and anti-inflammatory actions [15]. Consequently, the pharmacological blocking of the aforementioned receptors is not limited to effects on the bronchial diameter alone.

As demonstrated in the comprehensive review [16], a number of factors have been identified as being capable of affecting the density or functionality of receptors. Infectious diseases such as RSV have been demonstrated to reduce the number of β2AR, thereby facilitating bronchoconstriction. Further, patients with respiratory infection diseases, like asthma, may experience reduced resensitization of the receptor. In the context of COPD, ADRβ2 polymorphisms have been shown to be associated with disease severity and progression. Furthermore, the utilization of bronchodilator medications blocking β2AR exerts an influence on receptor mechanisms. In the aforementioned review [16], the authors analysed the findings of research that demonstrated that the chronic use of β2-adrenoceptor agonists induces tachyphylaxis and a rebound in airway hyperresponsiveness in patients diagnosed with both COPD and asthma. These circumstances result in diminished treatment effectiveness.

Given the shared aetiology of COPD and lung cancer, which is largely attributable to tobacco smoking, a strong association between the two diseases has been demonstrated [17]. It is evident that smoking has a significant impact on various pathological factors, including, but not limited to, growth factors, the activation of intracellular pathways, and epigenetics [18]. It has been demonstrated that COPD is an independent risk factor for lung cancer. As demonstrated by Young et al. [19], individuals who smoke and exhibit airflow obstruction are up to five times more prone to developing lung cancer than those without lung function impairment. Even when the overdiagnosis of COPD is excluded from the analysis, there is a doubled incidence of lung cancer in this patient group compared to the general population [20]. At the same time, there are currently no tools to verify spirometric obstruction in the presence of these two conditions. Therefore, there is a necessity to seek alternative diagnostic techniques that can supplement existing measurement methodologies. A search of the PubMed database for publications related to “airway obstruction detection” yielded almost twenty-nine thousand results. However, when ultrasonography was incorporated into the search process, the resulting number of results demonstrated a notable reduction of over fourfold. This paper aims to provide a comprehensive overview of the methods utilized for the identification of airway obstructions, with a focal emphasis on the use of lung ultrasonography (LUS) in the assessment of diaphragm movement impairments. Additionally, this study aims to delineate the inherent limitations of the discussed techniques.

## 2. Standard Methods for Detecting Airway Obstruction

### 2.1. Spirometry

Spirometry remains the diagnostic standard for the assessment of airway obstruction. As previously stated, the most recent GOLD guidelines stipulate that a decrease in the FEV1/FVC ratio to a level below 70% following the administration of a bronchodilator is considered indicative of obstruction [12]. Nevertheless, it has been demonstrated over time that this index is not optimal and can result in numerous misinterpretations. The FEV1/FVC ratio, which is employed to categorize degrees of obstruction in accordance with the GOLD guidelines as mild, moderate, and severe, may lead to an overdiagnosis of obstruction in older adult patients and an underdiagnosis in younger patients [21]. These considerations are reflected in the ATS/ERS guidelines, which currently recommend relating the results obtained to the Z-score [22]. These guidelines are supported by the Polish Pulmonary Society, which accepts the diagnosis of obstruction when the FEV1/FVC after the administration of a bronchodilator is reduced to less than the LLN rather than 70%, as maintained by the GOLD [23].

The utility of spirometry in the context of airway obstruction diagnosis has been corroborated in numerous scientific publications. In a study conducted on 235 patients presenting signs and symptoms of airway obstruction, the combination of spirometry with clinical and radiological results enabled the classification of the types of obstructive airway diseases, including asthma, ACOS (asthma–COPD overlap syndrome), chronic bronchitis, and emphysema [24]. These findings are consistent with those reported by Kitaguchi et al. [25], who observed differences in FEV1, the peak expiratory flow rate (PEF), and the residual volume (RV), and in response to short-acting β2-agonists between COPD, asthma, and ACOS patients with persistent airflow limitation. Even if spirometry is highly sensitive for predicting obstruction, with a sensitivity of 94%, the test also exhibits low specificity (24%) and a negative predictive value of 53% [26]. This indicates that, in many cases, obstruction may remain undetected. It is, therefore, evident that the underdiagnosis of COPD represents a significant clinical issue. A screening study conducted in Manchester enrolled a large cohort of smokers aged 55–74 in a spirometry test. Of these individuals, 37.4% exhibited evidence of airflow obstruction. Notably, half of those with airflow obstruction had no previous diagnosis of COPD despite the presence of characteristic symptoms. As estimated by the authors, one in ten of the screening attendees exhibited symptoms of previously unrecognized airflow obstruction. When considering the asymptomatic patients, 8.7% of them demonstrated evidence of airflow obstruction. Concomitantly, both groups showed an increased prevalence of morbidity and mortality [4]. Furthermore, diagnostic issues may arise in instances where spirometric values are borderline, indicating mild airway obstruction. In a cohort study on 8307 patients divided based on the GOLD stages, the addition of Parameter D to the spirometry, which is based on the shape of volume–time analysis, enabled the detection of patients with airflow obstruction in whom spirometry appeared to be normal according to traditional criteria [27]. In a separate recent study, the authors ascertained the diagnostic sensitivity to be 80.72% and the diagnostic specificity to be 94.5% in cases of the detection of obstructive airway disease based on the assessment of a decline in FEV1/FVC [28]. It is evident that spirometry is a test that is dependent on the subject’s effort, which renders it susceptible to a number of factors that may impact the results [29]. The ERS/ATS criteria indicate that a 10% alteration in FEV1 or FVC is noteworthy [22]. However, due to the dependence on the forced manoeuvres performed by the patient, the results may be inaccurate in the event of a less favourable day or an examination conducted in an inappropriate manner (for example, immediately following exercise, in slim-fit clothing, with cooperation difficulties in older adults) [29,30]. Furthermore, even in instances where approved references are available, there are exceptional cases where the predicted values are extrapolated beyond the limits of the underlying data. This is the approach used for patients over 70 years of age based on ECSC (European Coal and Steel Community) data and for patients over 80 years of age based on NHANES III (the third National Health and Nutrition Examination Survey) data, which may lead to errors [31,32]. Finally, it should be noted that results obtained on different devices and in different centres may differ significantly [29]. It seems, therefore, reasonable to posit that a crucial step in enhancing the health status of the general population is the assessment of airway obstruction through the utilization of diverse measurement techniques.

### 2.2. Body Plethysmography

An additional method for evaluating airway obstruction is body plethysmography. The test is principally based on the assessment of airway resistance (Raw) and its volume-related measures of specific Raw (sRaw) and specific airway conductance (sGaw), which serve to identify any obstructions to the flow of air. The sensitivity of this method is superior for the narrowing of extrathoracic or large central intrathoracic airways in comparison to those of more peripheral intrathoracic airways [22]. The findings of the study conducted by Barros et al. [33] indicate that high values of Raw (≥0.380 KPa/l/s) exhibited a sensitivity of 59.2% and a specificity of 92.7% in the detection of airway obstruction. Another study, which was conducted on children with asthma, demonstrated that the combination of body plethysmography and spirometry proved more effective than spirometry alone in the detection of airway obstruction [34]. These results are consistent with those of another study, in which only plethysmography was capable of discerning the distinctions between smokers without COPD and healthy controls, which were not identified by spirometry [35]. Similarly, in exercise-induced bronchoconstriction, the maximum changes observed in body plethysmography preceded those observed in spirometry [36]. Nevertheless, the test necessitates substantial cooperation from the patient and their confinement to a body plethysmography chamber, which renders the test considerably more challenging and precludes patients with claustrophobia from participating. What is more, body plethysmography remains a considerably less accessible and more costly tool than spirometry, which precludes its extensive utilization in the routine assessment of airway obstruction [37]. In addition to the aforementioned limitations, neither spirometry nor plethysmography has the capacity to diagnose unilateral obstruction, which may be caused by large bronchial narrowing, for example, in patients with lung cancer.

### 2.3. Oscillometry

An additional method of evaluating the presence and degree of airway obstruction is through oscillometric testing. According to available data, oscillometry has a higher sensitivity in the detection of small airway disease (SAD) compared to spirometry [38]. Additionally, the test itself is significantly more straightforward to conduct, as it does not necessitate forced exhalations. During the oscillometric test, which is conducted with tidal breaths, pressure impulses are applied to the airways at frequencies of 5, 11, and 19 Hz. The respiratory system’s response to these stimuli allows for the calculation of both its resistance and reactance. Researchers agree that oscillometric testing is preferred in patients who have difficulty performing spirometry testing, with a special focus on older adults and children <10 years of age, as well as patients with exacerbations of lung diseases such as COPD [30,39,40].

The assessment of airway obstruction in oscillometric testing is dependent on the utilization of resistance as an indicator of pressure loss along the course of the pressure pulse, extending deep into the airway. Consequently, it is indicative of the diameter of the bronchi. The information obtained at different frequencies allows for the verification of whether the obstruction found in the patient concerns all airways (5 Hz, R5), only the central airways (19 Hz, R19), or whether it is located in the peripheral airways (5–19 Hz, R5–19) [38,41]. A further parameter, reactance at 5 Hertz (X5), reflecting variations in volume in relation to the applied pressure, exhibits a lower value in cases of heterogeneity in the distribution of airway diameters. Such heterogeneity can be observed in instances of obstructive lung diseases, particularly when the collapse of small airways is present [28,38]. Oscillometric testing is a valuable addition to the assessment of lung function, as it correlates significantly with the results of other pulmonary function tests [42,43]. In patients with COPD, oscillometric impairment is found to correlate with the GOLD stage [44]. A further study indicated that it is feasible to diagnose COPD with a sensitivity of 79.1% and a specificity of 78.0% through the utilization of oscillometric results [45]. A recent multicentre study on 2459 patients determined a sensitivity of 66.7% and specificity of 81.5% in obstructive airway disease detection based on an R5 z-score increase [28]. A subsequent investigation revealed that R5 and R20 levels were notably elevated in patients diagnosed with asthma with airflow limitation when compared to those with COPD [25]. Further, elevated R5–20 demonstrated the presence of SAD in patients with a preserved FEV1/FVC ratio and decreased FEV1 in comparison to a control group of healthy subjects [46]. Nevertheless, due to the complexity of interpreting the results, the absence of standardized criteria and diagnostic algorithms, and the discrepancies in outcomes obtained using different measurement instruments, this technique remains relatively underutilized. As a result, its contribution to the assessment of airway obstruction remains significantly constrained.

### 2.4. Imaging Tests

Finally, a diagnosis of airway obstruction can be made through the use of imaging studies. The most prevalent and accessible imaging test with sufficient sensitivity to permit visualization of an airway obstruction is chest computer tomography (CT). A resolution range of 200 to 300 micrometres allows for a comprehensive evaluation of the seventh- to ninth-grade bronchus of the lung [47]. Among the morphological changes that may be observed in the context of evaluating SAD in obstructive diseases are the thickening of airway walls, the degree of lumen obstruction, the presence of inflammation, the development of fibrosis, and the occurrence of mucus blockages [48]. Furthermore, other components of obstructive diseases, such as emphysema and air trapping with mosaic perfusion, are evaluated through visual assessment [49,50]. Accordingly, research indicates that CT scans can identify abnormalities before the manifestation of clinical symptoms and prior to impairments in pulmonary function tests [51,52]. Similarly, in patients with asthma, the thickness of the airway wall, as determined by CT, was found to correlate with the severity of the disease [53]. Grenier et al. [54] concluded that the presence of bronchial wall thickening, bronchiectasis, and morphological abnormalities indicative of distal airway disease can be detected on CT scans in asthmatic patients. Niimi et al. [55] obtained analogous results, observing that the degree of airway obstruction, as determined by CT, in patients with asthma was associated with the duration and severity of the disease. The results obtained from a study of asthmatic patients indicated the presence of CT scan abnormalities in 80% of the subjects, predominantly characterized by bronchial wall thickening [56]. Concurrently, a correlation was observed between a decrease in the FEV1/FVC ratio and the thickness of the airway wall. A study demonstrated that participants with COPD can be identified with a high degree of accuracy, even using low-dose inspiratory and expiratory CT scans obtained for lung cancer screening (a sensitivity of 63% and a specificity of 88%) [57]. The utilization of CT airway features enables the differentiation of COPD from asthma with a diagnostic accuracy rate of 66% (a sensitivity of 73% and a specificity of 64%) [58]. Moreover, an increase in airway wall thickness of 1 mm has been demonstrated to correlate with an increase in annual disease exacerbation in patients diagnosed with COPD [59]. A comprehensive review of the extant literature reveals a significant correlation between pulmonary function test results and CT findings [60]. Given the favourable outcomes observed, the question arises as to whether CT can be incorporated into the standard diagnostic approach for airway obstruction. Nevertheless, the present examination is distinguished by a number of limitations, including, but not limited to, the radiation dose, its high costs, and its low availability [61].

A secondary imaging modality employed for the assessment of airway obstruction is magnetic resonance (MR). Despite the numerous challenges associated with imaging lung tissue, such as low spatial resolution, the presence of air within the lungs, and the air–tissue interfaces that result in magnetic microfields, the utilization of appropriate sequences and a polarized gas (typically helium) can yield high-quality images of lung tissue [62]. According to the results, MR fluoroscopy facilitates the differentiation between COPD patients and healthy individuals by diminishing the diaphragmatic excursion [63]. Hyperpolarized helium-3 MR has been demonstrated to be a valuable tool for predicting postbronchodilator reversibility in patients with mild-to-moderate asthma who have been stable for a period of 6 years [64]. This includes patients who have not experienced changes in lung function, medication, or exacerbations. Altes et al. [62] obtained analogous results, observing peripheral ventilation defects located at nonposterior sites. These defects were attributed to small-airway dysfunction in symptomatic asthma patients. The primary benefit of this test is its lack of radiation exposure. However, it is important to note that it is less accessible than CT, more time-consuming, and more expensive to perform, leaving the use of MR for assessing airway obstruction confined exclusively to research studies [50].

Ultimately, the visualization of airways can be achieved through the implementation of single-photon emission computed tomography (SPECT) and positron-emission tomography (PET). Both of these procedures are costly and complex, and they are associated with substantial radiation exposure. SPECT involves the inhalation of particulates, typically technegas (a radiological agent). The analysis of its distribution facilitates the visualization of the heterogeneous distribution of small-airway disease with tracer deposition in the upper airways during testing with methacholine [65]. Nonetheless, owing to the suboptimal spatial resolution and the inability to visualize only small airways, this technique is not suitable for the diagnosis of airway obstruction. Lastly, PET is a test that is frequently utilized in the evaluation of cancer advancement, indicating the activity of cells within various organs, including the lungs. An experimental PET application analyzing lung gas exchange, analogous to SPECT, utilizes an inhaled tracer (like nitrogen 13 gas) whose reduced accumulation in specific regions of the lungs signifies hypoventilation. Nevertheless, the spatial resolution of PET is approximately 1 cm, which is considerably lower than that of CT and MR [66]. Preliminary reports suggest the potential of the aforementioned method for assessing airway narrowing in patients with asthma, given the heterogeneity of distal airway dysfunction [67]. Despite their historical application, both techniques have not gained widespread popularity in the assessment of airway obstruction.

## 3. Materials and Methods

**Figure 1:**
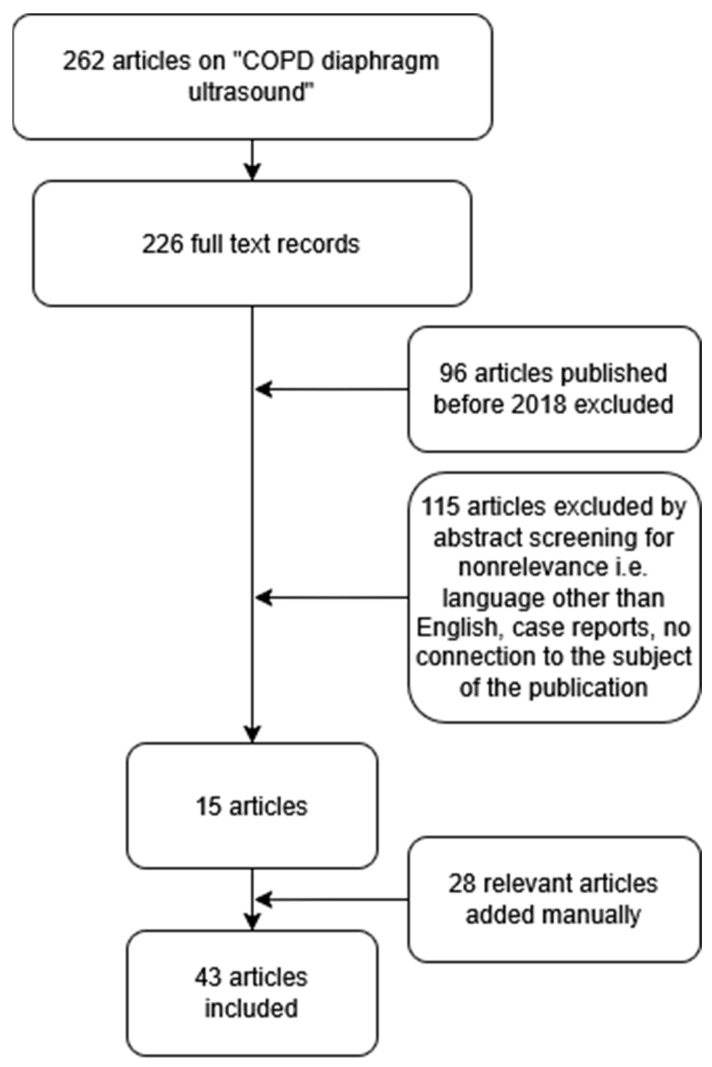
A flowchart of the literature selection process.

As previously stated, in the event of tumour lesions spreading in the airways, spirometry is unable to verify the basis of the airway obstruction identified. Consequently, in view of the diagnostic difficulties experienced by patients with lung cancer and established recent airway obstruction, there is a necessity to explore alternative approaches to diagnosis. The literature suggests that LUS has potential value in this area. Accordingly, we performed a comprehensive literature search in PubMed in February 2025 using the search algorithm “COPD” AND “diaphragm” AND “ultrasound”. Research focused on the application of ultrasonography in the detection and differentiation of COPD in adults. The investigators focused on the most recent (2018 and newer) and relevant publications by reading abstracts to select the studies. Case reports, papers written in languages other than English, publications with questionable methodology, and papers deemed as focusing on subjects irrelevant to the publication were excluded from the analysis. A number of relevant studies that were published before 2018 were manually added to the database. The flowchart is illustrated in Figure 1.

## 4. Ultrasonography

### 4.1. Lung Ultrasonography

Compared to the aforementioned lung function tests, LUS is a novel method for the assessment of respiratory disorders [68]. LUS interpretation consists of both real images and artefacts, which occur due to the difference in acoustic impedance between the chest wall and lung tissue. Analyzing successively in a well-aerated lung, the waves produced by the ultrasound machine are reverberated between the pleura and the transducer, creating horizontal artefacts known as A-lines [69]. The occurrence of these artefacts is a physiological phenomenon under the condition of present pleural sliding—the sign of normal backward and forward movement between visceral and parietal pleura during respiration [70]. If there is an excess of fluid in the interstitial space, the high impedance between the air in the alveoli and the pulmonary interstitium leads to the creation of vertical, comet tail-like artefacts, which are generated from the pleura line and extend to the bottom of the screen. These artefacts—B-lines—are associated with, but not limited to, pulmonary oedema [69,71]. B-lines may also be observed in the physiological context, although in small numbers, with no more than two from a longitudinal approach [72].

Additionally, LUS allows for the diagnosis of pneumothorax and pleural effusion with high sensitivity (78.6% and 94%, respectively) and specificity (98% in both conditions), which, paired with noninvasiveness and wide availability, further consolidates the method [73,74]. The increased understanding of artefacts produced by the interaction of ultrasound waves with lung airspaces led to the publication of the BLUE protocol, which is, to this date, used in the population of critically ill patients and allows rapid assessment of acute respiratory distress. In patients with acute respiratory failure, exacerbations of obstructive lung diseases can be diagnosed using the BLUE protocol. Predominant A-lines with concurrent lung sliding, which describes the correct movement between visceral and pulmonary pleura, indicate asthma or COPD as the underlying cause of deterioration [75,76]. Nevertheless, in cases of COPD exacerbations, LUS is typically restricted to the exclusion of other potential causes of acute respiratory failure, such as the aforementioned pulmonary oedema, pneumothorax, or pleural effusion [77].

On the other hand, ultrasonographic images of stable COPD may not differ significantly from those of normal lungs. Irregularities in the pleural line may be observed, although this sign is not exclusive to obstructive lung diseases [78]. As demonstrated by Sperandeo et al. [79], pleural line irregularities have been observed in all patients diagnosed with pulmonary fibrosis. Secondly, the prevalence of more prominent A-lines increased in 60% of COPD patients, whereas in the remaining 40%, the ultrasound image was normal [78]. Similar results came from a study by Esmaeel et al. [80], where 70% of patients with stable COPD showed more prominent A-lines compared to 15% in the control group. Additionally, a reduction of lung movement due to hyperinflation or the presence of subpleural bullae may mimic pneumothorax via a reduction in lung sliding signs [81,82]. In summary, there are no unambiguous and typical ultrasound features on lung imaging present only in patients with COPD, which could thus allow the diagnosis of obstructive disease. Conversely, the assessment of the diaphragm has been shown to be beneficial in this context.

### 4.2. Diaphragm Ultrasonography

**Figure 2:**
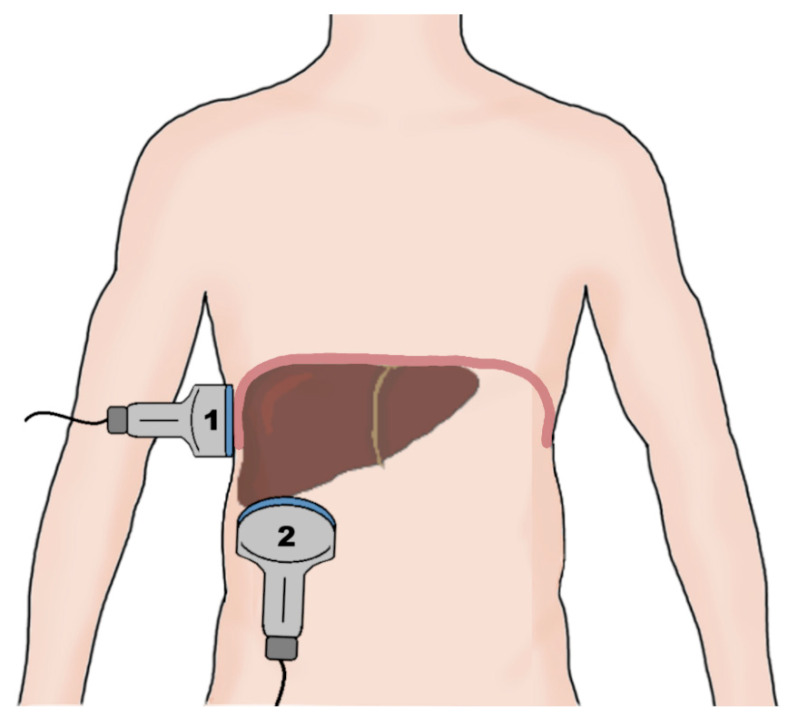
A schematic of the probe placement during an evaluation of the right hemidiaphragm. The diaphragm thickness is measured using a linear probe (1) placed at the zone of apposition of the diaphragm to the rib cage between the 8th and 9th intercostal spaces. The diaphragm excursion is measured using a convex probe (2) placed in the subcostal view, utilizing the liver as the sonographic window during respiratory movement of the diaphragm.

Diaphragmatic ultrasound is a noninvasive and quick method of assessment of diaphragm mobility, with intraclass correlation coefficients ranging from 88% to 99% for the intraobserver agreement [83]. The ultrasonographic examination of diaphragm function consists of measuring diaphragmatic mobility and diaphragm thickness. The mobility of the diaphragm is usually assessed using a convex probe in the subcostal view during quiet breathing, voluntary sniffing, and deep breathing using the liver and the spleen as acoustic windows in the M-mode [84]. Diaphragmatic thickness evaluation is performed in patients in a supine or sitting position at the zone of apposition from the diaphragm to the rib cage between the eighth and ninth intercostal spaces using a linear probe (Figure 2) [85].

**Figure 3:**
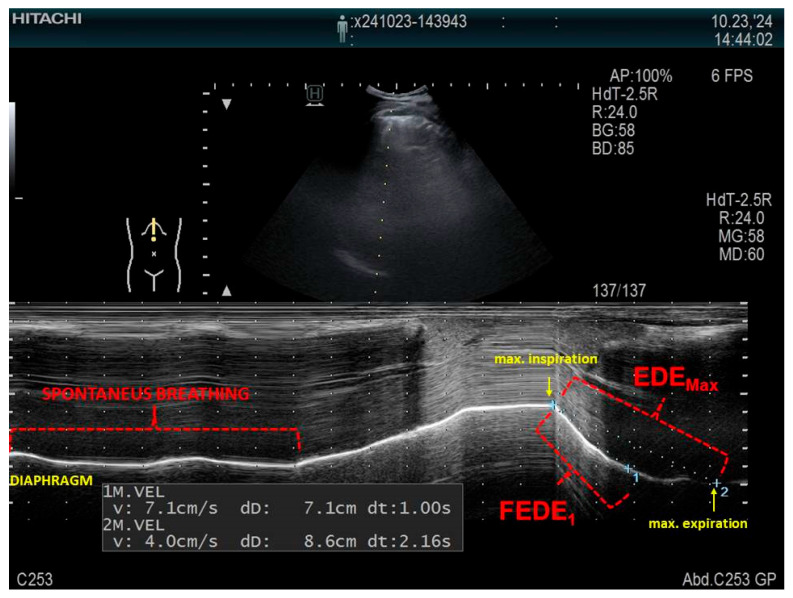
An example of measuring the mobility of the right hemidiaphragm using a convex probe in the M-mode in a patient in a supine position, using the liver as the acoustic window. FEDE_1_, the ratio of the diaphragmatic excursion in the first second of forced expiration; EDE_Max_, the maximum expiratory diaphragmatic excursion.

The examination allows for measurements of the diaphragmatic thickness (Tdi), the thickening fraction (TF), and the ratio between diaphragm thickness at the end of spontaneous breathing and diaphragm thickness at maximal inspiration (ΔTmax) [86]. An example of measuring the mobility of the right hemidiaphragm is presented in Figure 3. LUS allows for bilateral examination, which is not possible using spirometry or oscillometry, and may prove useful in the diagnosis of unilateral obstruction, which may be caused by lung cancer, among other conditions.

The utilization of ultrasound in the evaluation of airway obstruction, measured by diaphragm movement dysfunction, has been documented in a limited number of reports. In patients with COPD, increased airway resistance, increased airflow restriction, and dynamic hyperinflation lead to the impairment of diaphragmatic function [87]. In a study conducted by Zanforlin et al. [88], the ratio of diaphragmatic excursion in the first second of forced expiration (FEDE1) to maximum expiratory diaphragmatic excursion (EDEMax), called the M-mode index of obstruction (MIO), which describes the speed of diaphragm relaxation, was a highly specific and sensitive marker of obstruction, with a cutoff point of 77%. By contrast, in a study conducted by Paulin et al. [89], diaphragmatic mobility was evaluated indirectly in the B-mode as the craniocaudal displacement of the intrahepatic branches of the portal vein. However, regardless of the ultrasound technique, COPD patients similarly presented less diaphragmatic mobility compared to controls (36.27 ± 10.96 mm vs. 46.33 ± 9.46 mm), especially subjects presenting greater air trapping, expressed as a percentage of the predicted residual volume (266.20 ± 55.30 vs. 209.74 ± 48.49), which correlated positively with lower exercise tolerance, as measured by the 6 min walk test. As concluded by Dos Santos Yamaguti et al. [90], a reduction in diaphragmatic mobility in COPD patients is strongly correlated with parameters quantifying air trapping, with an R-value of −0.60 for the residual volume and −0.76 for the residual volume-to-total lung capacity ratio. In addition, the results of a study conducted by Qaiser et al. [91] reported a strong correlation between the reduced diaphragmatic excursion in the COPD group (2.39 ± 0.92 cm vs. 4.18 ± 0.58 cm) and the FEV1/FVC ratio (an R-value of 0.85). Moreover, Silva et al. [92] showed a correlation between diaphragmatic mobility during deep inspiration and predicted FEV1 (r = 0.36), predicted RV (r = −0.42), RV/TLC (r = −0.61), and the distance reached in a 6 min walk test (r = 0.46). Patients with a modified Medical Research Council (mMRC) score < 2 exhibited greater diaphragmatic excursion than patients with a score ≥ 2 (mean difference = 13.20  ±  4.6 mm).

The comparison of the diaphragmatic excursion of the right hemidiaphragm and the lung ultrasound score in the assessment of lung hyperinflation in patients with stable COPD was the subject of a study by Chen et al. [93]. The researchers scanned the anterior, lateral, and posterior chest walls in 72 intercostal spaces, dividing the chest walls into 16 different regions. In each of the intercostal spaces, the researchers observed the presence or absence of lung sliding signs. Additionally, pleural sliding displacement (PSD) was measured, defined as the distance between the lung and liver junction at the end of inhalation and the end of expiration, with a normal value of <12 mm for males and <10 mm for females. The lung ultrasound score depended on the PSD value and the number of lung areas showing the disappearance of lung sliding signs, with values ranging from 0 points in patients with normal values of PSD and normal pleural sliding to 5 points in patients with low PSD and the disappearance of pleural sliding in more than 12 lung regions. The statistical analysis showed a positive correlation of the lung ultrasound score with the RV, total lung capacity (TLC), RV/TLC, and functional residual capacity (FRC) and a negative correlation with the inspiratory capacity (IC) and IC/TLC. The correlation was stronger than between diaphragm excursion and the corresponding pulmonary function tests.

The influence of pharmacotherapy on the diaphragmatic kinetics in patients with COPD was the subject of a study by Wangüemert-Pérez et al. [94]. The researchers found that the 3-month use of indacaterol/glicopyrronium significantly improved diaphragmatic mobility and the minimum and maximum diaphragm thickness. A significant negative correlation was detected between diaphragmatic mobility and the GOLD staging of COPD patients using FEV1 in a study by Youssuf et al. [78]. Similar results were achieved in a study by Scheibe et al. [95], in which the researchers compared direct right hemidiaphragm ultrasonography with the sonographic up- and downward movement of the lung silhouette. In addition to finding a strong correlation between the two methods, the median distance of the right hemidiaphragm movement was found to correlate with COPD severity. Furthermore, Evrin et al. [96] conducted a study evaluating COPD severity using diaphragm ultrasound conducted by three different methods: the aforementioned lung silhouette method on both sides of the chest in a scapular line (Lung Sil Right and Lung Sil Left), the right-side B-mode evaluation of diaphragm movement using the liver as a sonographic window from the anterior position, measured as the distance between the highest and lowest point of the right hemidiaphragm during forced inhalation and exhalation (Ant B-Mode Right), and the bilateral examination of hemidiaphragms recorded in the M-mode (Ant M-Mode Right and Ant M-Mode Left). The researchers found a strong correlation between FEV1 and Lung Sil Right, Lung Sil Left, Ant B-Mode Right, and Ant M-Mode Right. Moreover, negative correlations were described between the annual number of exacerbations and Lung Sil Right (r = −0.599) and the annual number of exacerbations and Ant M-Mode Right (r = −0.587). No significant differences were detected between the lung silhouette method and the anterior method.

Additionally, An et al. [97] showed the possibility of using diaphragm ultrasound as a biomarker of the acute exacerbation of COPD (AECOPD). In the study, the researchers divided COPD patients into exacerbation (acute change of symptoms requiring medication change) and stable groups (patients not requiring systemic corticosteroids or antibiotics due to AECOPD for 3 months). The exacerbation group had a lower body mass index (20.9 vs. 24.2), lower diaphragm thickening fraction (TFmax) (94.8 ± 8.2% vs. 158.4 ± 83.5%), and lower diaphragmatic excursion (DEmax) (30.8 ± 11.1 mm vs. 40.5 ± 12.5 mm) compared to the stable group. ROC curve analyses were performed to distinguish the AECOPD group with a TFmax cutoff value of 93.8% (a sensitivity of 68.4% and a specificity of 78.8%) and a DEmax value of 44.9 mm (a sensitivity of 95.2% and a specificity of 44.8%). After dividing the patients into groups with TFmax and DEmax above and below the cutoff point, the researchers found that the low TFmax and low DEmax groups exhibited poorer lung function and a higher proportion of AECOPD. After adjusting for age, sex, BMI, and comorbidities, both low TFmax and low DEmax were associated with AECOPD, with odds ratios of 8.40 and 11.51, respectively.

Furthermore, there is an absence of consensus regarding the thickness of the diaphragm in patients diagnosed with COPD. Baria et al. [98] reported no significant differences in the diaphragm thickness and thickening ratio between the control and COPD groups. By contrast, Esmaeel et al. [80] found a positive correlation between diaphragmatic thickness and mobility with FEV1 and FVC. However, the differences in these measurements were statistically insignificant between the GOLD groups. Yalçın et al. [99] described a significant reduction in diaphragm thickness during deep inspiration (53.44 vs. 71.99 mm) and forced expiration (47.70 vs. 79.47 mm), as well as mobility (50.03 vs. 76.43 mm) in COPD patients compared to controls. However, the COPD group consisted mainly of the GOLD group D patients (64%), with no analysis performed between the GOLD groups. The results of a study by Topcuoğlu et al. [100] showed a significant bilateral reduction in thickness, thickening fraction, and mobility in the COPD group compared to healthy adults. Moreover, using the GOLD criteria to divide patients into mild (FEV1 ≥ 80%), moderate (50% ≤ FEV1 < 80%), and severe (30% ≤ FEV1 < 50%) groups, the researchers found that the left and right diaphragm thickness at forced exhalation, as well as the right diaphragm thickness at total lung capacity, were significantly higher in mild COPD than in moderate COPD and in moderate COPD than in severe COPD. It is, however, important to note the small group of patients enrolled in the study: 11 with mild COPD, 13 with moderate COPD, and 6 with severe COPD.

A study by Ramachandran et al. [101] compared the diaphragmatic thickness and mobility and the rectus femoris cross-sectional area (RFCSA) between COPD patients and healthy controls. The RFCSA was measured using a linear ultrasonographic probe placed perpendicularly to the long axis of the thigh, three-fifths of the distance from the anterior superior iliac spine to the superior patellar border, with the subject in a supine position and the leg in passive extension and further calculated planimetrically in the B-mode. In the studied group, the diaphragm thickness (1.8 ± 0.5 mm vs. 2.2 ± 0.6 mm) and the RFCSA (4.8 ± 1.3 cm vs. 6.12 ± 1.2 cm) were significantly lower in the COPD group, indicating both diaphragmatic and skeletal muscle dysfunction. The diaphragm mobility, however, was not significantly lower between the groups. Moreover, as concluded by Okura et al. [102], the thickness of the diaphragm at TLC and the change ratio between the diaphragm’s thickness at TLC and RV is significantly impaired in COPD patients in comparison to both younger and older healthy adults. However, no significant differences were found between the groups in the measurements of diaphragm thickness at a functional residual capacity and residual volume. Additionally, a study by Schulz et al. [103] performed on COPD patients confirmed a significant correlation between FEV1 and diaphragm mobility, as well as between FEV1 and the diaphragm thickening fraction. Lung function parameters reflecting respiratory muscle strength—maximal respiratory pressure (Pimax) and the ratio of airway occlusion pressure to maximal respiratory pressure (P0.1/Pimax)— also correlated with the diaphragmatic excursion. Furthermore, a significant negative correlation was found between diaphragmatic mobility and both residual volume and diaphragmatic thickness. Finally, no correlation was found between diaphragmatic thickness and FEV1, Pimax, and P0.1/Pimax. By contrast, Ogan et al. [104] measured the diaphragmatic thickness in COPD patients and healthy subjects during tidal volume and deep inspiration on both sides of the diaphragm. The researchers found no correlation between both groups in all of the measurements. There was also no significant difference between the diaphragmatic thickness and COPD severity, respiratory function, and frequency of exacerbations. However, it is important to note that the RV and TLC measurements were not available for the researchers to prove the presence of hyperinflation. Moreover, Rittayamai et al. [105] compared diaphragm thickness at the end of inspiration and expiration, excursion during maximum isometric inspiratory effort against a one-way valve (DE-max), also known as the Muller manoeuvre, and the thickening fraction during tidal breathing (TFdi-tidal) and during the Muller manoeuvre (TFdi-max) between COPD patients and healthy controls. The researchers also evaluated the diaphragm force reserve ratio, calculated as 1—(TFdi-tidal/TFdi-max), representing the diaphragmatic reserve relative to the maximum capacity of the diaphragm. The results revealed that TFdi-tidal was significantly higher in the COPD group compared to controls (30.8% vs. 21.1%), with 40.7% in patients suffering from severe COPD according to the GOLD criteria. TFdi-max and DE-max were significantly lower in the COPD group (65.3% vs. 84.3% for TFdi-max and 3.9 vs. 5.0 cm for DE-max). The diaphragm force reserve ratio was lower in patients with COPD than in control subjects, and it fell with the increase in the GOLD staging. During the 2-year follow-up, patients with COPD exacerbations had a significantly lower diaphragm reserve ratio compared to subjects without exacerbation (0.46 vs. 0.55).

Additionally, Lim et al. [106] investigated the changes in the diaphragmatic function during acute exacerbation of COPD. The researchers measured the thickening fraction and diaphragmatic excursion of both hemidiaphragms within the first 72 h of exacerbation and during the stable phase. The percent change in diaphragmatic thickness between end expiration and end inspiration (DTF) on the right side was significantly decreased during COPD exacerbation compared to the stable phase of COPD. The decrease in left DTF was not statistically significant. The diaphragmatic excursion on both sides did not show significant differences between the acute exacerbation and stable phases. Moreover, the change in diaphragmatic excursion between the stable and exacerbation periods was positively correlated with the time to the next exacerbation and negatively correlated with time needed to recover from the exacerbation. The main limitation of the study was, however, the small sample size, consisting of 10 patients.

Until now, there have been no reports analysing diaphragmatic obstruction in the ultrasound evaluation of patients with vaginosis and its use to verify the above-mentioned diagnosis, especially in the case of concomitant overlaps with other disease entities that may impair airway patency, e.g., massive lymphadenopathy or proliferative changes in the bronchial tree. In this context, ultrasonography has demonstrated considerable potential. It is, therefore, evident that further studies are required in order to verify the aforementioned area of LUS application. A comparison of the methods used for detecting airway obstruction is presented in Table 1.

### 4.3. The Utilization of Ultrasound Assessment of the Diaphragm in Clinical Practice

**Figure 4:**
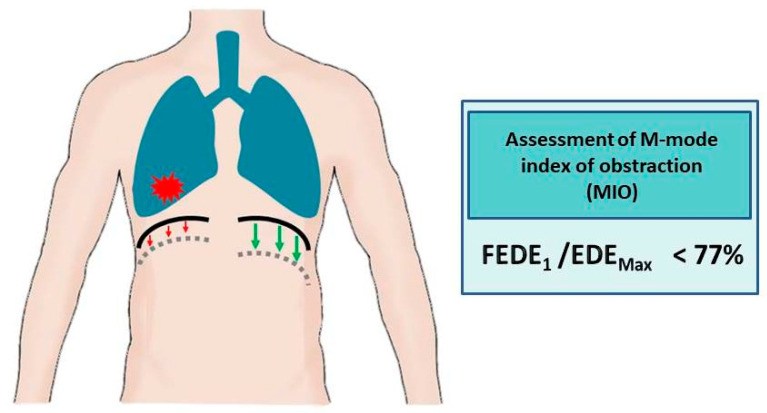
Airway obstruction in the ultrasound assessment of the diaphragm (green arrows indicate normal diaphragmatic movements, red arrows illustrate reduced diaphragm movements caused by tumour masses). FEDE_1_, ratio of the diaphragmatic excursion in the first second of forced expiration; EDE_Max_, maximum expiratory diaphragmatic excursion.

**Figure 5:**
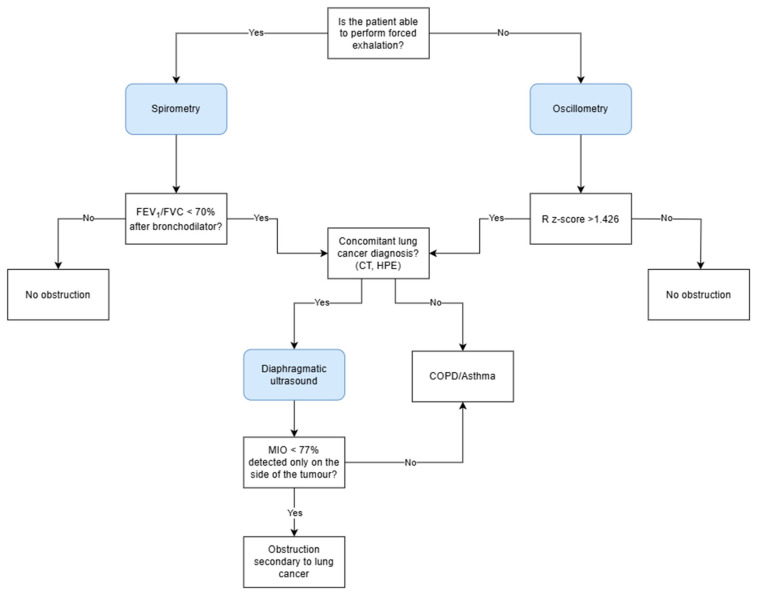
The diagnostic algorithm for the assessment of airway obstruction in patients without previously diagnosed obstructive disease. FEV_1_, forced expiratory volume during the first second; FVC, forced vital capacity; R, resistance; MIO, M-mode index of obstruction; COPD, chronic obstructive pulmonary disease.

In accordance with the extensive evidence base that has been established for the utilization of diaphragm ultrasound in the evaluation of airway obstruction, there is considerable potential for its incorporation into routine clinical practice, particularly within pulmonology and oncology settings and primary care. In patients without a prior diagnosis of obstructive airway disease (COPD/asthma) or in cases where such a diagnosis has been established based on spirometry results within the last three months, and where a concomitant diagnosis of lung cancer has been made, it is recommended that an ultrasound examination be performed to rule out unilateral obstruction. A CT scan of the thorax, performed as a component of the diagnostic and therapeutic evaluation of lung cancer, facilitates the evaluation of regions exhibiting impaired airway flow, heterogeneous ventilation, or atelectatic areas, including those resulting from the presence of cancerous lesions. It is then recommended to undertake an ultrasound examination with the transducer placed on the right and left under the costal arch, with an inclination of approximately 30°, in order to visualize the cranio-caudal excursion of the diaphragm. In order to calculate the MIO, it is necessary to assess the diaphragmatic mobility in one second of forced expiration and between maximal inhalation and exhalation. An MIO index of less than 77% has been shown to be indicative of airway obstruction. The finding of the aforementioned in isolation, only in the area of neoplastic lesions, will serve to strengthen the suspicion of obstruction secondary to infiltrative masses and not due to obstructive airway disease (Figure 4). In this group of patients, under medical supervision, it is possible to verify the diagnosis of COPD and, if deemed necessary, to discontinue bronchodilator drugs. The proposal for the diagnostic algorithm is illustrated in Figure 5.

## 5. Limitations of Ultrasonography

**Figure 6:**
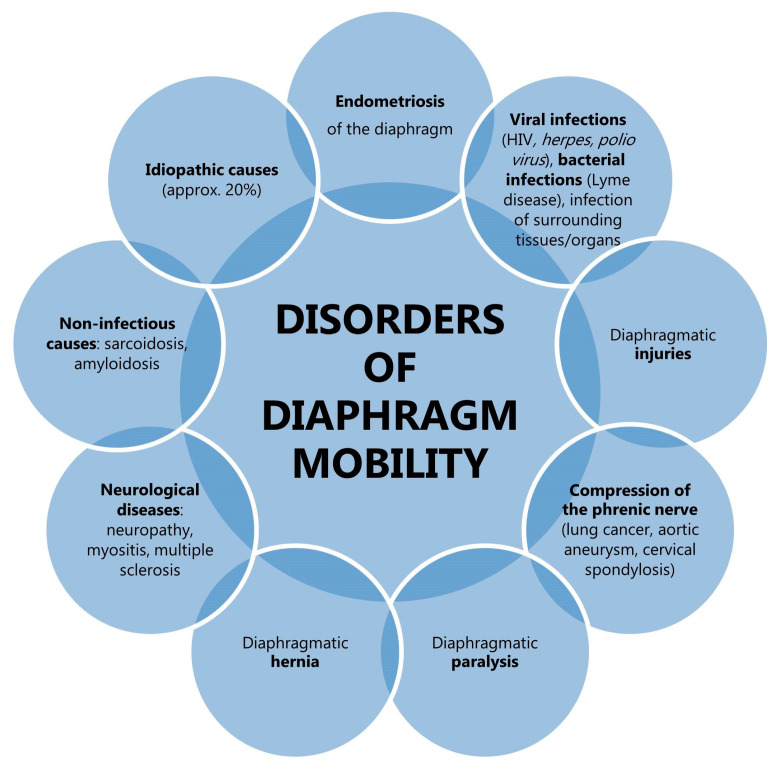
Examples of diseases that have the potential to affect the mobility of the diaphragm.

As the ultrasonographic images of normal lungs are similar to the lungs of patients with obstructive respiratory disease, the utility of lung ultrasonography in the diagnosis of obstruction remains uncertain, apart from the differential diagnosis of sudden respiratory distress. Diaphragm sonography proves to be a promising method of assessment of obstruction. It is not, however, without limitations. Visibility of the left hemidiaphragm in ultrasonographic examination may be difficult due to the small size of the splenic sonographic window. As demonstrated in the findings reported by Boussuges et al. [84], the diaphragmatic movement of the left hemidiaphragm during deep breathing was only possible to record in 21% of subjects. However, a study by Suga et al. [107] using dynamic breathing MR presented similar mobility of the left and right hemidiaphragms in 68% of patients diagnosed with advanced pulmonary emphysema. Therefore, the presence of diaphragm mobility disorders does not necessarily imply a pathological cause. It is also important to note that diaphragmatic dysfunction may be a consequence of a number of factors, including paralysis, infections, trauma, systemic diseases, or compression (Figure 6) [108]. A further critical limiting factor is the dependence of the diaphragm examination result on the experience of the examiner. The consensus of the experts concluded that diaphragm ultrasonography in critically ill patients can be considered an easy skill to learn, with a steep learning curve. Conversely, the measurement of diaphragm thickness is considered a more difficult skill to acquire, with a slow learning curve, and a minimum of 40 examinations is required for independent use of the method in daily practice [109]. The outcome of the examination also relies on the position of the patient, with greater diaphragmatic excursion for the same inspired volume in subjects in a supine position [110]. Finally, as with spirometry, the result of the evaluation depends on the patient’s cooperation since the diaphragmatic excursion relies on the maximal inspiratory effort of the subject [83]. In view of the frequently advanced age of patients, the presence of underlying lung disease and concomitant illnesses, or communication difficulties, compliance is often found to be inadequate.

## 6. Conclusions

Ultrasonography remains a promising area of research in the detection of obstruction. The possibility of the diagnosis of unilateral pathology, broad availability, relatively low cost and short time of examination, and the feasibility of the assessment of patients in both supine and erect positions may establish sonography as a complementary examination method to other methods for the diagnosis of obstruction. It is, however, important to note the limitations of ultrasonography, including difficulty in the examination of the left hemidiaphragm and the requirement of experience of the researcher. Nevertheless, further research is needed to consolidate ultrasonography in this field of research.

## Figures and Tables

**Table 1:** A comparison of the selected methods used to detect airway obstruction.

Method	Sensitivity	Specificity	Advantages	Disadvantages
Spirometry	80.72% [28]	94.5% [28]	Easily accessible Low cost Standardized diagnostic criteria	Measurement during forced-breathing manoeuvres Numerous contraindications
Oscillometry	66.7% [28]	81.5% [28]	High sensitivity in SAD diagnosis Measurement during spontaneous breathing	Low availability
				Complex interpretation Lack of standardized criteria
Computer tomography	62.7% [57]	87.9% [57]	High correlation with pulmonary function test results	Low availability High cost
				Exposure to ionizing radiation
Diaphragm ultrasound	83.33% [88]	96.61% [88]	Noninvasiveness Possibility of detection of unilateral obstruction	Experienced investigator required
				Low accessibility of left hemidiaphragm during examination Measurement during forced-breathing manoeuvres Numerous conditions may impair diaphragm mobility

SAD, small airway disease.

## Data Availability

The original contributions presented in this study are included in the article. Further inquiries can be directed to the corresponding author.

## References

[1] Barnes P.J., Burney P.G., Silverman E.K., Celli B.R., Vestbo J., Wedzicha J.A., Wouters E.F. (2015). Chronic obstructive pulmonary disease. Nat. Rev. Dis. Primers.

[2] Burgel P.R. (2011). The role of small airways in obstructive airway diseases. Eur. Respir. Rev..

[3] Buist A.S., McBurnie M.A., Vollmer W.M., Gillespie S., Burney P., Mannino D.M., Menezes A.M., Sullivan S.D., Lee T.A., Weiss K.B. (2007). International variation in the prevalence of COPD (the BOLD Study): A population-based prevalence study. Lancet.

[4] Balata H., Harvey J., Barber P.V., Colligan D., Duerden R., Elton P., Evison M., Greaves M., Howells J., Irion K. (2020). Spirometry performed as part of the Manchester community-based lung cancer screening programme detects a high prevalence of airflow obstruction in individuals without a prior diagnosis of COPD. Thorax.

[5] Morrison C.B., Markovetz M.R., Ehre C. (2019). Mucus, mucins, and cystic fibrosis. Pediatr. Pulmonol..

[6] Rogers D.F. (2007). Physiology of airway mucus secretion and pathophysiology of hypersecretion. Respir. Care.

[7] Pederiva F., Rothenberg S.S., Hall N., Ijsselstijn H., Wong K.K.Y., von der Thüsen J., Ciet P., Achiron R., Pio d′Adamo A., Schnater J.M. (2023). Congenital lung malformations. Nat. Rev. Dis. Primers.

[8] Boogaard R., Huijsmans S.H., Pijnenburg M.W., Tiddens H.A., de Jongste J.C., Merkus P.J. (2005). Tracheomalacia and bronchomalacia in children: Incidence and patient characteristics. Chest.

[9] Barnes P.J., Szefler S.J., Reddel H.K., Chipps B.E. (2019). Symptoms and perception of airway obstruction in asthmatic patients: Clinical implications for use of reliever medications. J. Allergy Clin. Immunol..

[10] Brady M.F., Burns B. Airway Obstruction. https://www.ncbi.nlm.nih.gov/books/NBK470562/.

[11] Hurst J.R., Skolnik N., Hansen G.J., Anzueto A., Donaldson G.C., Dransfield M.T., Varghese P. (2020). Understanding the impact of chronic obstructive pulmonary disease exacerbations on patient health and quality of life. Eur. J. Intern. Med..

[12] Venkatesan P. (2024). GOLD COPD report: 2024 update. Lancet Respir. Med..

[13] Fiore M., Ricci M., Rosso A., Flacco M.E., Manzoli L. (2023). Chronic Obstructive Pulmonary Disease Overdiagnosis and Overtreatment: A Meta-Analysis. J. Clin. Med..

[14] Sator L., Horner A., Studnicka M., Lamprecht B., Kaiser B., McBurnie M.A., Buist A.S., Gnatiuc L., Mannino D.M., Janson C. (2019). Overdiagnosis of COPD in Subjects With Unobstructed Spirometry: A BOLD Analysis. Chest.

[15] Bai T.R. (1992). Beta 2 adrenergic receptors in asthma: A current perspective. Lung.

[16] Manti S., Gambadauro A., Galletta F., Ruggeri P., Piedimonte G. (2024). Update on the Role of β2AR and TRPV1 in Respiratory Diseases. Int. J. Mol. Sci..

[17] Durham A.L., Adcock I.M. (2015). The relationship between COPD and lung cancer. Lung Cancer.

[18] Barnes P.J., Adcock I.M. (2011). Chronic obstructive pulmonary disease and lung cancer: A lethal association. Am. J. Respir. Crit. Care Med..

[19] Young R.P., Hopkins R.J. (2010). Link between COPD and lung cancer. Respir. Med..

[20] Young R.P., Duan F., Chiles C., Hopkins R.J., Gamble G.D., Greco E.M., Gatsonis C., Aberle D. (2015). Airflow Limitation and Histology Shift in the National Lung Screening Trial. The NLST-ACRIN Cohort Substudy. Am. J. Respir. Crit. Care Med..

[21] Thomas E.T., Glasziou P., Dobler C.C. (2019). Use of the terms “overdiagnosis” and “misdiagnosis” in the COPD literature: A rapid review. Breathe.

[22] Stanojevic S., Kaminsky D.A., Miller M.R., Thompson B., Aliverti A., Barjaktarevic I., Cooper B.G., Culver B., Derom E., Hall G.L. (2022). ERS/ATS technical standard on interpretive strategies for routine lung function tests. Eur. Respir. J..

[23] Sliwiński P., Górecka D., Jassem E., Pierzchała W. (2014). Polish respiratory society guidelines for chronic obstructive pulmonary disease. Pneumonol. Alergol. Pol..

[24] Ozkaya S., Dirican A., Tuna T. (2016). The objective evaluation of obstructive pulmonary diseases with spirometry. Int. J. Chron. Obstruct. Pulmon. Dis..

[25] Kitaguchi Y., Yasuo M., Hanaoka M. (2016). Comparison of pulmonary function in patients with COPD, asthma-COPD overlap syndrome, and asthma with airflow limitation. Int. J. Chron. Obstruct. Pulmon. Dis..

[26] Loh C.H., Genese F.A., Kannan K.K., Lovings T.M., Peters S.P., Ohar J.A. (2018). Spirometry in Hospitalized Patients with Acute Exacerbation of COPD Accurately Predicts Post Discharge Airflow Obstruction. Chronic. Obstr. Pulm. Dis..

[27] Bhatt S.P., Bhakta N.R., Wilson C.G., Cooper C.B., Barjaktarevic I., Bodduluri S., Kim Y.I., Eberlein M., Woodruff P.G., Sciurba F.C. (2018). New Spirometry Indices for Detecting Mild Airflow Obstruction. Sci. Rep..

[28] Liang X., Zheng J., Gao Y., Zhang Z., Han W., Du J., Lu Y., Chen L., Wang T., Liu J. (2022). Clinical application of oscillometry in respiratory diseases: An impulse oscillometry registry. ERJ Open Res..

[29] Liou T.G., Kanner R.E. (2009). Spirometry. Clin. Rev. Allergy Immunol..

[30] Czajkowska-Malinowska M., Tomalak W., Radliński J. (2013). Quality of Spirometry in the Elderly. Adv. Respir. Med..

[31] Quanjer P.H., Tammeling G.J., Cotes J.E., Pedersen O.F., Peslin R., Yernault J.C. (1993). Lung volumes and forced ventilatory flows. Eur. Respir. J..

[32] Hankinson J.L., Odencrantz J.R., Fedan K.B. (1999). Spirometric reference values from a sample of the general U.S. population. Am. J. Respir. Crit. Care Med..

[33] Barros R., Raposo L., Oliveira A.S., Barbara C. (2021). Airway resistance and characterization of airway obstruction. Eur. Respir. J..

[34] Korten I., Zacharasiewicz A., Bittkowski N., Hofmann A., Lex C. (2019). Asthma control in children: Body plethysmography in addition to spirometry. Pediatr. Pulmonol..

[35] Gupta Y.S., Shah S.S., Ahire C.K., Kamble P., Khare A.S., More S.S. (2018). Body plethysmography in chronic obstructive pulmonary disease patients: A cross-sectional study. Lung India.

[36] Schulze J., Smith H.J., Eichhorn C., Salzmann-Manrique E., Dreßler M., Zielen S. (2019). Correlation of spirometry and body plethysmography during exercise-induced bronchial obstruction. Respir. Med..

[37] Hanlon P. Spirometry and Plethysmography: Combining Diagnostic Power. Respir. Ther..

[38] Brashier B., Salvi S. (2015). Measuring lung function using sound waves: Role of the forced oscillation technique and impulse oscillometry system. Breathe.

[39] Domínguez-Martín C., Cano A., Díez-Monge N., Alonso-Rubio A.M., Pérez-García I., Arroyo-Romo M.T., Casares-Alonso I., Barbero-Rodríguez A.M., Grande-Alvarez R., Martínez-Rivera M.T. (2023). Spirometry and respiratory oscillometry: Feasibility and concordance in schoolchildren with asthma. Pediatr. Pulmonol..

[40] Terraneo S., Rinaldo R.F., Sferrazza Papa G.F., Ribolla F., Gulotta C., Maugeri L., Gatti E., Centanni S., Di Marco F. (2021). Distinct Mechanical Properties of the Respiratory System Evaluated by Forced Oscillation Technique in Acute Exacerbation of COPD and Acute Decompensated Heart Failure. Diagnostics.

[41] Smith H.J., Reinhold P., Goldman M.D. (2005). Forced Oscillation Technique and Impulse Oscillometry. Eur. Respir. J..

[42] D’Ascanio M., Viccaro F., Calabrò N., Guerrieri G., Salvucci C., Pizzirusso D., Mancini R., De Vitis C., Pezzuto A., Ricci A. (2020). Assessing Static Lung Hyperinflation by Whole-Body Plethysmography, Helium Dilution, and Impulse Oscillometry System (IOS) in Patients with COPD. Int. J. Chron. Obstruct. Pulmon. Dis..

[43] Vink G.R., Arets H.G., van der Laag J., van der Ent C.K. (2003). Impulse oscillometry: A measure for airway obstruction. Pediatr. Pulmonol..

[44] Crim C., Celli B., Edwards L.D., Wouters E., Coxson H.O., Tal-Singer R., Calverley P.M., ECLIPSE investigators (2011). Respiratory system impedance with impulse oscillometry in healthy and COPD subjects: ECLIPSE baseline results. Respir. Med..

[45] Chaiwong W., Namwongprom S., Liwsrisakun C., Pothirat C. (2020). Diagnostic Ability of Impulse Oscillometry in Diagnosis of Chronic Obstructive Pulmonary Disease. COPD.

[46] Zhao N., Wu F., Peng J., Zheng Y., Tian H., Yang H., Deng Z., Wang Z., Li H., Wen X. (2022). Preserved ratio impaired spirometry is associated with small airway dysfunction and reduced total lung capacity. Respir. Res..

[47] Lindenmaier T.J., Kirby M., Paulin G., Mielniczuk L., Cunningham I.A., Mura M., Licskai C., Parraga G. (2016). Pulmonary artery abnormalities in ex-smokers with and without airflow obstruction. COPD.

[48] Hogg J.C., Paré P.D., Hackett T.L. (2017). The Contribution of Small Airway Obstruction to the Pathogenesis of Chronic Obstructive Pulmonary Disease. Physiol. Rev..

[49] Ostridge K., Wilkinson T.M. (2016). Present and future utility of computed tomography scanning in the assessment and management of COPD. Eur. Respir. J..

[50] Laurent F., Tunon de Lara M. (2011). Assessment of imaging techniques for evaluating small-airway disease in asthma. Rev. Mal. Respir..

[51] Li T., Zhou H.P., Zhou Z.J., Guo L.Q., Zhou L. (2021). Computed tomography-identified phenotypes of small airway obstructions in chronic obstructive pulmonary disease. Chin. Med. J..

[52] Maarsingh H., Bidan C.M., Brook B.S., Zuidhof A.B., Elzinga C., Smit M., Oldenburger A., Gosens R., Timens W., Meurs H. (2019). Small airway hyperresponsiveness in COPD: Relationship between structure and function in lung slices. Am. J. Physiol. Lung Cell Mol. Physiol..

[53] Eddy R.L., Svenningsen S., Kirby M., Knipping D., McCormack D.G., Licskai C., Nair P., Parraga G. (2020). Is Computed Tomography Airway Count Related to Asthma Severity and Airway Structure and Function?. Am. J. Respir. Crit. Care Med..

[54] Grenier P., Mourey-Gerosa I., Benali K., Brauner M.W., Leung A.N., Lenoir S., Cordeau M.P., Mazoyer B. (1996). Abnormalities of the airways and lung parenchyma in asthmatics: CT observations in 50 patients and inter- and intraobserver variability. Eur. Radiol..

[55] Niimi A., Matsumoto H., Amitani R., Nakano Y., Mishima M., Minakuchi M., Nishimura K., Itoh H., Izumi T. (2000). Airway wall thickness in asthma assessed by computed tomography. Relation to clinical indices. Am. J. Respir. Crit. Care Med..

[56] Gupta S., Siddiqui S., Haldar P., Raj J.V., Entwisle J.J., Wardlaw A.J., Bradding P., Pavord I.D., Green R.H. (2009). Brightling CE. Qualitative analysis of high-resolution CT scans in severe asthma. Chest.

[57] Mets O.M., Buckens C.F., Zanen P., Isgum I., van Ginneken B., Prokop M., Gietema H.A., Lammers J.W., Vliegenthart R., Oudkerk M. (2011). Identification of chronic obstructive pulmonary disease in lung cancer screening computed tomographic scans. JAMA.

[58] Moslemi A., Kontogianni K., Brock J., Wood S., Herth F., Kirby M. (2022). Differentiating COPD and asthma using quantitative CT imaging and machine learning. Eur. Respir. J..

[59] Han M.K., Kazerooni E.A., Lynch D.A., Liu L.X., Murray S., Curtis J.L., Criner G.J., Kim V., Bowler R.P., Hanania N.A. (2011). Chronic obstructive pulmonary disease exacerbations in the COPD Gene study: Associated radiologic phenotypes. Radiology.

[60] Bakker J.T., Klooster K., Vliegenthart R., Slebos D.J. (2021). Measuring pulmonary function in COPD using quantitative chest computed tomography analysis. Eur. Respir. Rev..

[61] Fred H.L. (2004). Drawbacks and limitations of computed tomography: Views from a medical educator. Tex. Heart Inst. J..

[62] Altes T.A., Powers P.L., Knight-Scott J., Rakes G., Platts-Mills T.A., de Lange E.E., Alford B.A., Mugler J.P., Brookeman J.R. (2001). Hyperpolarized 3He MR lung ventilation imaging in asthmatics: Preliminary findings. J. Magn. Reason. Imaging.

[63] Unal O., Arslan H., Uzun K., Ozbay B., Sakarya M.E. (2000). Evaluation of diaphragmatic movement with MR fluoroscopy in chronic obstructive pulmonary disease. Clin. Imaging.

[64] Eddy R.L., Svenningsen S., Licskai C., McCormack D.G., Parraga G. (2019). Hyperpolarized Helium 3 MRI in Mild-to-Moderate Asthma: Prediction of Postbronchodilator Reversibility. Radiology.

[65] Pellegrino R., Biggi A., Papaleo A., Camuzzini G., Rodarte J.R., Brusasco V. (2001). Regional expiratory flow limitation studied with Technegas in asthma. J. Appl. Physiol..

[66] Musch G., Venegas J.G. (2005). Positron emission tomography imaging of regional pulmonary perfusion and ventilation. Proc. Am. Thorac. Soc..

[67] Tgavalekos N.T., Tawhai M., Harris R.S., Musch G., Vidal-Melo M., Venegas J.G., Lutchen K.R. (2005). Identifying airways responsible for heterogeneous ventilation and mechanical dysfunction in asthma: An image functional modeling approach. J. Appl. Physiol..

[68] Buda N., Mendrala K., Skoczyński S., Pasquier M., Mazur P., Garcia E., Darocha T. (2023). Basics of Point-of-Care Lung Ultrasonography. N. Engl. J. Med..

[69] Bhoil R., Ahluwalia A., Chopra R., Surya M., Bhoil S. (2021). Signs and lines in lung ultrasound. J. Ultrason..

[70] Miller A. (2016). Practical approach to lung ultrasound. BJA Educ..

[71] Gargani L. (2011). Lung ultrasound: A new tool for the cardiologist. Cardiovasc. Ultrasound.

[72] Dietrich C.F., Mathis G., Blaivas M., Volpicelli G., Seibel A., Wastl D., Atkinson N.S., Cui X.W., Fan M., Yi D. (2016). Lung B-line artefacts and their use. J. Thorac. Dis..

[73] Alrajab S., Youssef A.M., Akkus N.I., Caldito G. (2013). Pleural ultrasonography versus chest radiography for the diagnosis of pneumothorax: Review of the literature and meta-analysis. Crit. Care.

[74] Yousefifard M., Baikpour M., Ghelichkhani P., Asady H., Shahsavari Nia K., Moghadas Jafari A., Hosseini M., Safari S. (2016). Screening Performance Characteristic of Ultrasonography and Radiography in Detection of Pleural Effusion; A Meta-Analysis. Emergency.

[75] Lichtenstein D.A., Mezière G.A. (2008). Relevance of Lung Ultrasound in the Diagnosis of Acute Respiratory Failure. Chest.

[76] Lichtenstein D.A. (2014). Lung ultrasound in the critically ill. Ann. Intensive Care.

[77] Rogoza K., Kosiak W. (2016). Usefulness of lung ultrasound in diagnosing causes of exacerbation in patients with chronic dyspnea. Pneumonol. Alergol. Pol..

[78] Youssuf H.A.A., Abdelnabi E.A., Abd El Hafeez A.M., Fathallah W.F., Ismail J.H. (2016). Role of transthoracic ultrasound in evaluating patients with chronic obstructive pulmonary disease. Egypt. J. Bronchol..

[79] Sperandeo M., Varriale A., Sperandeo G., Filabozzi P., Piattelli M.L., Carnevale V., Decuzzi M., Vendemiale G. (2009). Transthoracic ultrasound in the evaluation of pulmonary fibrosis: Our experience. Ultrasound Med. Biol..

[80] Esmaeel H.M., Atta K.A., Khalaf S., Gadallah D. (2024). Clinical Utility of Chest Sonography in Chronic Obstructive Pulmonary Disease Patients Focusing on Diaphragmatic Measurements. Tuberc. Respir. Dis..

[81] Aziz S.G., Patel B.B., Ie S.R., Rubio E.R. (2016). The Lung Point Sign, not Pathognomonic of a Pneumothorax. Ultrasound Q..

[82] Slater A., Goodwin M., Anderson K.E., Gleeson F.V. (2006). COPD Can Mimic the Appearance of Pneumothorax on Thoracic Ultrasound. Chest.

[83] Santana P.V., Cardenas L.Z., de Albuquerque A.L.P., Carvalho C.R.R., Caruso P. (2020). Diaphragmatic ultrasound: A review of its methodological aspects and clinical uses. J. Bras. Pneumol..

[84] Boussuges A., Gole Y., Blanc P. (2009). Diaphragmatic motion studied by m-mode ultrasonography: Methods, reproducibility, and normal values. Chest.

[85] Carrillo-Esper R., Pérez-Calatayud Á.A., Arch-Tirado E., Díaz-Carrillo M.A., Garrido-Aguirre E., Tapia-Velazco R., Peña-Pérez C.A., Espinoza-de Los Monteros I., Meza-Márquez J.M., Flores-Rivera O.I. (2016). Standardization of Sonographic Diaphragm Thickness Evaluations in Healthy Volunteers. Respir. Care.

[86] Boussuges A., Rives S., Finance J., Chaumet G., Vallée N., Risso J.J., Brégeon F. (2021). Ultrasound Assessment of Diaphragm Thickness and Thickening: Reference Values and Limits of Normality When in a Seated Position. Front. Med..

[87] Kakhaki H.E.S., Alesaeidi S., Siri G., Arya A., Sarafraz H., Otadi K., Yazdi N.A., Abedinzadeh K. (2023). Diaphragmatic Ultrasound Advantages in Chronic Obstructive Pulmonary Disease (COPD) Patients: A Systematic Review and Metaanalysis. Ethiop. J. Health Sci..

[88] Zanforlin A., Smargiassi A., Inchingolo R. (2014). Ultrasound analysis of diaphragm kinetics and the diagnosis of airway obstruction: The role of the M-mode index of obstruction. Ultrasound Med. Biol..

[89] Paulin E., Yamaguti W.P.S., Chammas M.C., Shibao S., Stelmach R., Cukier A., Carvalho C.R. (2007). Influence of diaphragmatic mobility on exercise tolerance and dyspnea in patients with COPD. Respir. Med..

[90] Dos Santos Yamaguti W.P., Paulin E., Shibao S., Chammas M.C., Salge J.M., Ribeiro M., Cukier A., Carvalho C.R. (2008). Air trapping: The major factor limiting diaphragm mobility in chronic obstructive pulmonary disease patients. Respirology.

[91] Qaiser M., Khan N., Jain A. (2020). Ultrasonographic Assessment of Diaphragmatic Excursion and its Correlation with Spirometry in Chronic Obstructive Pulmonary Disease Patients. Int. J. Appl. Basic Med. Res..

[92] Figueira Silva B.C., Abreu D.C., Souza Y.R., Figueiredo M., Macêdo J.F., Mafort T.T., Rufino R., Costa C.H.D. (2024). Ultrasonography as a way of evaluating the diaphragm muscle in patients with chronic obstructive pulmonary disease. Medicine.

[93] Chen Y., Li J., Zhu Z., Lyu G. (2024). Lung Ultrasound Assessment of Lung Hyperinflation in Patients with Stable COPD: An Effective Diagnostic Tool. Int. J. Chron. Obstruct. Pulmon. Dis..

[94] Wangüemert-Pérez A.L., Figueira-Gonçalves J.M., Ramallo-Fariña Y., Guanche-Dorta S., Golpe R. (2023). Ultrasound assessment of diaphragmatic dynamics in patients with chronic obstructive pulmonary disease after treatment with indacaterol/glycopyrronium. Rev. Clin. Esp..

[95] Scheibe N., Sosnowski N., Pinkhasik A., Vonderbank S., Bastian A. (2015). Sonographic evaluation of diaphragmatic dysfunction in COPD patients. Int. J. Chron. Obstruct. Pulmon. Dis..

[96] Evrin T., Korkut S., Ozturk Sonmez L., Szarpak L., Katipoglu B., Smereka J., Guven R., Akpinar E.E. (2019). Evaluating Stable Chronic Obstructive Pulmonary Disease by Ultrasound. Emerg. Med. Int..

[97] An T.J., Yoo Y.J., Lim J.U., Seo W., Park C.K., Rhee C.K., Yoon H.K. (2022). Diaphragm Ultrasound is an Imaging Biomarker that Distinguishes Exacerbation Status from Stable Chronic Obstructive Pulmonary Disease. Int. J. Chron. Obstruct. Pulmon. Dis..

[98] Baria M.R., Shahgholi L., Sorenson E.J., Harper C.J., Lim K.G., Strommen J.A., Mottram C.D., Boon A.J. (2014). B-Mode Ultrasound Assessment of Diaphragm Structure and Function in Patients With COPD. Chest.

[99] Yalçın B., Sekmenli N., Baktık B., Bekçi T.T. (2022). Evaluation of diaphragm thickness and function with ultrasound technique and comparison with spirometry in stable chronic obstructive pulmonary disease. Tuberk. Toraks..

[100] Topcuoğlu C., Yümin E.T., Hizal M., Konuk S. (2022). Examination of diaphragm thickness, mobility and thickening fraction in individuals with COPD of different severity. Turk. J. Med. Sci..

[101] Ramachandran P., Devaraj U., Patrick B., Saxena D., Venkatnarayan K., Louis V., Krishnaswamy U.M., D’souza G.A. (2020). Ultrasonographic assessment of skeletal muscle mass and diaphragm function in patients with chronic obstructive pulmonary disease: A case-control study. Lung India.

[102] Okura K., Iwakura M., Shibata K., Kawagoshi A., Sugawara K., Takahashi H., Satake M., Shioya T. (2020). Diaphragm thickening assessed by ultrasonography is lower than healthy adults in patients with chronic obstructive pulmonary disease. Clin. Respir. J..

[103] Schulz A., Erbuth A., Boyko M., Vonderbank S., Gürleyen H., Gibis N., Bastian A. (2022). Comparison of Ultrasound Measurements for Diaphragmatic Mobility, Diaphragmatic Thickness, and Diaphragm Thickening Fraction with Each Other and with Lung Function in Patients with Chronic Obstructive Pulmonary Disease. Int. J. Chron. Obstruct. Pulmon. Dis..

[104] Ogan N., Aydemir Y., Evrin T., Ataç G.K., Baha A., Katipoğlu B., Süzen B., Akpınar E.E. (2019). Diaphragmatic thickness in chronic obstructive lung disease and relationship with clinical severity parameters. Turk. J. Med. Sci..

[105] Rittayamai N., Chuaychoo B., Tscheikuna J., Dres M., Goligher E.C., Brochard L. (2020). Ultrasound Evaluation of Diaphragm Force Reserve in Patients with Chronic Obstructive Pulmonary Disease. Ann. ATS.

[106] Lim S.Y., Lim G., Lee Y.J., Cho Y.J., Park J.S., Yoon H.I., Lee J.H., Lee C.T. (2019). Ultrasound Assessment of Diaphragmatic Function During Acute Exacerbation of Chronic Obstructive Pulmonary Disease: A Pilot Study. Int. J. Chron. Obstruct. Pulmon. Dis..

[107] Suga K., Tsukuda T., Awaya H., Takano K., Koike S., Matsunaga N., Sugi K., Esato K. (1999). Impaired respiratory mechanics in pulmonary emphysema: Evaluation with dynamic breathing MRI. J. Magn. Reson. Imaging.

[108] Kokatnur L., Vashisht R., Rudrappa M. Diaphragm Disorders. StatPearls Publishing.

[109] Haaksma M.E., Smit J.M., Boussuges A., Demoule A., Dres M., Ferrari G., Formenti P., Goligher E.C., Heunks L., Lim E.H.T. (2022). Expert consensus On Diaphragm UltraSonography in the critically ill (EXODUS): A Delphi consensus statement on the measurement of diaphragm ultrasound-derived parameters in a critical care setting. Crit. Care.

[110] Houston J.G., Angus R.M., Cowan M.D., McMillan N.C., Thomson N.C. (1994). Ultrasound assessment of normal hemidiaphragmatic movement: Relation to inspiratory volume. Thorax.

